# Testimony of an Ethiopian girl sexually assaulted by her stepfather: a case report

**DOI:** 10.1186/s13256-018-1777-x

**Published:** 2018-08-26

**Authors:** Yohannes Mehretie Adinew, Beza Tamirat Mekete, Yimenu Mehretie Adinew

**Affiliations:** 1 College of Health Sciences and Medicine, Wolaita Sodo University, Sodo, Ethiopia; 2 College of Medicine and Health Science, Jigjiga University, Jigjiga, Ethiopia; 3 College of Public Health and Medical Sciences, Jimma University, Jimma, Ethiopia

**Keywords:** Incest, Intrafamilial child sexual abuse, Child sexual abuse, Stepfather, Ethiopia

## Abstract

**Background:**

The sensitiveness and stigma associated with sexual assault deter many victims from speaking about their experiences. This silence of victims worsens the problem, especially in patriarchal communities like Ethiopia where sex is taboo and girls are encouraged to remain behind curtains.

**Case presentation:**

This is the personal testimony of a 25-year-old Amhara woman, a student at an Ethiopian public university, and it is presented in her own words. The data were collected during an in-depth interview on 19 April 2015. The interview was conducted in a private environment and her name was concealed to protect her anonymity. A digital voice recorder was used to audio-tape the interview which was later transcribed and translated verbatim from the local language, Amharic, to English.

**Conclusions:**

The trouble and pain our participant experienced is beyond description. Four themes emerged from her narrative: Incest assault, repeated assault, feelings of guilt and shame, and orphanhood. Incest is both more common and more severe in stepparent families but reported cases are only the very tip of the iceberg and thus may greatly under-represent actual population rates. Therefore, more effort is required to hold the perpetrators accountable and restore the life of the victims.

## Background

Sexual violence against women continues to be a public health problem. It has a devastating impact on women’s lives. The Secretary-General of the United Nations (UN) claimed that it was a particularly unkind human rights violation that must be eradicated [1].

The sensitiveness and stigma associated with sexual assault deter many victims from speaking about their experiences [2]. A culture of sexual discrimination against girls is common in patriarchal communities like Ethiopia, where girls are supposed to stay indoors and serve males [3]. As a result, most perpetrators will not be held accountable.

Children from single-parent families and stepfamilies have a higher risk of victimization compared to those living with two biological parents [4]. The greatest risk is from family perpetrators, stepparents [5, 6]. Child rape and molestation continue to contribute to the major health problems of many girls in Ethiopia; as victims they are more likely to have a major depressive episode, posttraumatic stress disorder, agoraphobia, social phobia, and sexual disorders [7].

Sexual abuse is a major problem with serious long-term negative consequences. Raped women require specific health services: psychological support, prevention of unwanted pregnancy, sexually transmitted infections including human immunodeficiency syndrome (HIV), and management of injuries [8]. However, public health services for these women are of varied quality in Ethiopia. The scarcity of resources leads to limited options for service provision.

The short-term and long-term sequelae of child sexual abuse (CSA) are complex; a clear understanding of the sequelae is helpful in planning treatment and directing allocation of scarce resources. Thus, the aim of this case report is to present one girl’s testimony, in her own words, of her experience of rape and suffering at the hands of her stepfather.

## Case presentation

This is the personal testimony of a 25-year-old Amhara woman, a final year student at an Ethiopian public university, and it is presented in her own words. The data were collected during an in-depth interview on 19 April 2015. The interview was audio-taped using a digital voice recorder. The main topics covered were her personal experience as a victim of incest rape, communities’ attitude, and the stigma and shame she faced as a victim. The interview was transcribed and translated verbatim from the local language, Amharic, to English. The transcript and translated version of the document were cross-checked with the original interview by an experienced sociologist.

It was another normal day in my office; I was analyzing data I had collected a week earlier on sexual violence from female university students when I heard a knock on my door. After I gave permission to enter, a frightened woman in her twenties entered. I greeted her and asked if I could help. “*Last week you said we can visit your office if we have a story to share on sexual assault*. *So, I came to tell what my stepfather did to me when I was young, only if you can make it anonymous and agree to use it after I graduate*,” she said. I agreed to her preconditions and gave her my word. Then she told me her story, the secret that has darkened her life, after a week of dilemma.

She was very nervous; she was not sure where to begin her story. I told her to take time and comfort herself first, and after taking a deep breath she started narrating, “…*My mom was a young, hardworking, single mother. I was her only child as my father passed away when I was only two. Life was very tough for us; she had to work a double shift for us to survive. We lived alone for six years then she started to see a humble guy. A year later he moved in and changed our life remarkably as he was gainfully employed. I was very happy to see a smile on my mom’s face; she even upgraded her education and got her bachelor degree and became a professional nurse. I was also transferred to a better school and my academic performance increased. It was a joy to have a supportive father and a caring mother.”*

*“Our happiness didn’t last long. Things started to change as my mom continued to spend more nights at work. I heard them arguing every single night as my father was jealous of her contacts with her coworkers and the way she dressed. That humble and caring father totally changed and made drinking his day-to-day habit. He started coming home late and even sometimes spent the night out. It was up to me to sit and wait for him especially when my mom was working during the night on the night shift at the hospital. Thus, I couldn’t sleep until he got home. Sometimes I was up all the night because he was chaotic and making annoying sounds. He took out his anger by breaking any material he found, but he never touched me as I was obedient and always took care of him. My sleepless and stressful nights made it difficult to actively attend my class. As a result, my school performance declined dramatically. Even though I was disappointed with my school results and our messy life, I never complained as I thought it was the only way I could help mom. Thus, I tried to cope with things, but I had never suspected that there could be worse things in life until that night.”*

### Incest assault

She remembers the day that put a big scar on her life and made her feel disabled with a lot of sadness and rage; she has cried her entire life. After sobbing for a while, she continued, “…*that night he came home after midnight blind drunk as usual. I was all alone as my mom was out for a night shift at work. He stood and stared at me like never before; I was terrified by the way he looked at me, but tried to calm him down and serve him dinner. Still staring at me, he said, ‘My today’s dinner is you.’ I didn’t have a clue what that meant. I giggled and walked to my bedroom. Minutes later, I heard a cracking sound, it was my bedroom door. There my stepfather stood stark naked and asked me if I was ready to have sex with him. I was deeply shocked to see him like that. It was a total nightmare. How in the world does a father talk to his daughter like that? I remember that my body was shaking and I was hoping he would leave, but that didn’t happen. He suddenly jumped onto my bed, throttled me, tore my dress and removed everything I was wearing including my underwear. I was only fifteen by then; only fifteen. I was confused and couldn’t even figure out what was going on. Let alone shouting or fighting back I couldn’t even breathe as I was suffocated. Then Heeee ra…ped me* (she cried for a while and stared at the tape recorder, her eyes were full of revenge and I could see every disappointment in her)*, hmm he enjoyed every bit of my pain and left me in tears in my mom’s home. I cried the whole night; I felt like there was dirt all over my body that could never be cleansed. I wished it to be a dream but it was not. Early next morning, he came to my room and told me to wash my body and the bed sheet before mom returns. I was amused and angry at the same time by his statement; I thought that because he did it when he was drunk he would never talk to me again and even would not have the guts to look me in the eyes when he is sober again. But to the contrary, he warned me to swallow my pain and go to school saying, ‘What happened last night remains between us, never tell your mom, don’t even think about it. If you fail to keep your mouth shut, I will destroy both of you. I will make your life hell.’”*

*“…I will never forget the day I had at school that day. I was disturbed, worn out, upset and worried the whole day. I couldn’t even sit properly because of the pain, let alone pay attention to class. I couldn’t enjoy my lunch and communicate with my friends. The worst part was I had to act normal while my world was upside down. The endless questions of my friends increased my tension and I lost confidence to go home after school. Had it been another day I would have been eager to see my mom, as she spends the night on her duty and I go to school before she arrives home. That day, that day was totally different, I was not the girl mom knew before. How could I look in her eyes? I felt like I had betrayed her and let her down. So, I decided not to go home, rather disappear. I even told myself that life had no meaning for me and I didn’t deserve to live anymore. How could I live with him under the same roof after all he has done to me? But, what about my mom, the poor woman who lived for her daughter, I was her only inspiration and she has no life without me. I understood that my foolish decision would shorten her life and I went home. She treated me as usual, warm hug and affectionate kisses. Even though I was relieved to see her bright smile, I couldn’t take it so I cried. She was startled and asked me, ‘What happened?’ Part of me wanted to tell her everything while the other part kept reminding me of the evil words he said. So I told her it was just because of a fight I had had with my best friend. She smiled and kissed me again and told me that everything would be fine and she would help me to make up with my friend. I said yes mom, everything will be fine, but it hasn’t been, since then. That night I was outraged to see him acting normal, as if nothing had happened. He tried to be the same father in front of my mom, but when mom went to the kitchen I told him to keep his distance from me. But, he told me to shut my silly mouth. I still couldn’t figure out how a man who was humble and whom I once trusted and loved as if he were my biological father turned out to be a monster.”*

### Repeated assault

*“Four days later my mom was on her night duty again and he came home early. I gave him water for his hands and served dinner; he asked me to eat with him but I refused. I couldn’t control my temper when he said ‘take it easy’ so I left him there and went to my room; he followed me and started arguing. He choked my throat when I asked him to leave my room. This time I didn’t give up easily, I tried to fight back but he smashed my head against the wall and I collapsed in his hands, then he raped me again on my own bed. I cried the whole night. I really missed my mom, this wouldn’t have happened to me if she were home. I lost hope in my future. I hated myself and decided to take my life by my hands. I knew that only death could give me a break from this animal. But my poor mom came to my mind whenever I thought of bad things. So I told myself to be strong.”*

*“The next day I told mom to change her job and spend the night with us, but she didn’t get my point and said, ‘My dear I am doing this for you. As you can see I am ageing now but you will need more money when you join university and I have to save as much money as I can. I don’t want my only daughter to face money issues.’ I wanted her to know the truth behind my request, but couldn’t find the guts to tell her and ruin her life. But the worst thing was my stepfather made it his habit to rape me every time my mom was on her night duty; hearing his footsteps near my room became a nightmare I had to face over and over again. He even started to treat me as if I were his mistress. He even compared me with my mom, saying he enjoyed having sex with me more than that old woman, my mom. I hated those dark days. I had even prayed to God to take me to him and never wake me up…”*

*“This miserable life of mine continued for months, but one morning I vomited in the middle of class; my friends took me out and gave me water. I had no one to discuss my problem with; I was continually losing weight and my grade was also declining. One of my best friends mocked that I was pregnant; she was right, it had been two months since I missed my period. I went home and told him straight away; he was not surprised and took me to a private clinic next morning when my friends were going to school. I aborted when I was supposed to be in class. He was not sorry for what I was undergoing because of him, he even told the clinician to give me long-term contraception so that I would not find myself in the same situation again. I was heartbroken to see how mean he was, I believed he was my guardian, but he raped me. I had accepted and treated him like my biological father, but he played with my life. What tore my heart more was the health professional, he didn’t seem to care a bit about me; he did only what my perpetrator asked him to do. How on earth did he not even ask me a single question when he saw me in school uniform? What should I expect? After all he is a man! They kept me in the clinic until students were released from school, so that I could walk home with them as if I were in the school. That night I couldn’t help mom with the housework as usual and told her that I was on my period and she comforted me.”*

### Feelings of guilt and shame

*“The next day he came to my room to have sex with me, as mom was not home. I decided to kill him with a knife I prepared earlier, but it didn’t work. He took away the knife and struggled with me to remove my underwear. Then I shouted and he left my room; when our neighbors came and knocked on the door he told them it was an accident and they should go home. The next morning on my way to school, one of our neighbors, mom’s best friend, was very curious and asked me to tell her what happened last night. I tried to control myself and said nothing but I couldn’t hide my tears. She was shocked to see me like that and took me to her house and asked me to tell her everything, promising she would not tell anyone including my mom. I couldn’t take it any longer and told her from the beginning. She couldn’t wait for me to finish, she ran to our home with an axe to kill him. He ran away when he saw her and she chased him until she was stopped by people. She was so very nervous that she forgot her promise and told them everything. I was humiliated by what happened. I was frozen like a tomb, unable to do or say anything. My life changed from that day on, I faced the worst stigma a girl of my age couldn’t bear. I was an agenda for the big and small, everybody talked about me. I dropped out from school because everyone in the school, including my close friends, was busy gossiping about my private life. Some male students even asked me to make out with them saying I had nothing to lose anymore. No one tried to help me, including the teachers, rather they made fun of me; some even accused me of seducing my mother’s husband.”*

### Orphanhood

She suddenly started crying loudly, I gave her as much time as she desired to calm herself down and asked her what had happened then and she continued, *“…unfortunately my mom arrived home when everything was in a mess and everybody was talking about it. Hearing the heartbreaking news she collapsed and never woke up again, though she was taken to hospital. I never got the chance to say goodbye to her and ask for forgiveness for keeping this secret for so long and making her find out about it that way. That day, I lost my glimmer of hope for once and for all. She was the reason I was able to bear all the pain, but my decision cost her life. She was the only hope I had; I didn’t spill a bean despite my suffering because I knew she would not withstand it with her cardiac condition. I was devastated to mourn my loss and face everybody with all the shame, humiliation, loneliness and guilt deep inside me. At a glance, I lost everything, I became an orphan. I had nowhere to go and no one to support me. But, thanks to God, my mom’s best friend took me in but it was difficult for me to blend in. I didn’t feel free to ask for my basic necessities; I didn’t want to be a burden on her family. I was awake most of the night because of the nightmare; I feel he is standing somewhere in my room watching me, I had a fear he would come and choke me. I dropped all my friends because I didn’t trust anyone. Back then, the only thing I wanted in life was to complete my education, join university and make true my mom’s dream.”*

*“…He took away from me everything I had: my childhood, my hope, above all my beloved mom. Despite the fact that he should be sentenced and serve time in jail, my criminal stepfather escaped justice and his whereabouts is unknown. No one stood for my rights and I was too financially and morally broken to bring the case to court.”* She wiped her tears and continued after a long sigh, “…*am still living with the pain and scar; I believe someday God will punish him the way he deserves. He made me hate all men; I can’t marry and start a family. How can I trust men after all that has happened to me by a person I had once loved and trusted? I can’t even imagine it. I don’t want any girl to experience my pain in her life; that is why I decided to share my story leaving behind all the discomforts. Single mothers should be careful to bring men to their daughters’ house.”*

## Discussion

Unwelcome sexual advances are a tense event that many children experience. Women do not always discuss such experiences or report them to the police. The person making the advances is not always a stranger, but can be a known person or even a family member. Such experiences can occur anytime in a woman’s life even as a child [9].

Most CSA is perpetrated by those known to the victims. Women with a history of CSA are more likely than non-abused women to originate from single-parent families or families with a high level of marital conflict [10]. In addition, these families are generally characterized by substance abuse and violence among parents [11].

Incestuous experience involving a father-figure is likely to be more traumatic than abuse by others for numerous reasons. Abuse by a parent involves greater betrayal and loss of trust than abuse by others [10, 12]. It may also reflect a significant level of family disturbance along with less available emotional support for the girl. There may also be a variety of other severe consequences, such as open conflict and family break-up. It is also probable that abuse by fathers or stepfathers is more likely to be of longer duration and with greater frequency than abuse by nonrelatives [10]. When stepfathers abuse their daughters, they are much more likely than any other relative to abuse them at the most serious level [13]. That is why stepfather to daughter incest is rated as the most traumatic [14] and has the worst outcomes [15] with long-lasting effects [16]. Stepfathers are more likely to use force or threats of force than are fathers, and abuse by stepfathers is more likely to include penetration than abuse by fathers [10].

The short-term and long-term sequelae of CSA are complex and a clear understanding of the effects is helpful in planning treatment and directing allocation of scarce resources. CSA is a causal agent in many negative adulthood outcomes, ranging from depression [17–19] and posttraumatic stress disorder [20–22], to life-threatening behaviors such as suicide ideation and suicide attempts [23–27]. Women with a history of CSA are also characterized by symptoms of sexual disturbance including fear of men, less sexual interest, and less sexual pleasure [28]. The trauma is certain to be severe if the abuse is at a younger age [12]. CSA also has a corrosive effect on self-esteem, making these women obvious targets for sexually exploitative men [14].

The duration and frequency of the abuse have an impact on its outcome; longer duration has greater trauma [29]. Repeated episodes or long duration of violent sexual assault results in severer outcomes, such as self-blame and depression [16]. Greater long-term harm is linked with abuse involving stepfather to daughter incest as it is expected to last long [10, 16] and that is why anxiety is more common among intrafamilial CSA victims than extrafamilial abuse victims [30]. The impact of these experiences manifests itself in negative feelings about men, sex, or self, and personalized feelings of anxiety [16].

The level of trauma and harm is also determined by the type of abuse sustained. For instance, contact CSA is more likely than noncontact abuse to result in a major depressive episode; sexual abuse involving penetration is more traumatic than abuse not involving penetration [12], which is evidenced by the higher prevalence of major depression among sexually abused women [31]. Women reporting CSA require treatment, usually for depression [10, 27, 30, 31].

Rape-related pregnancy is a significant problem that needs closer attention. Although the total number of rape-related pregnancies may account for only a small portion of unintended pregnancies, the occurrence of pregnancy resulting from rape or incest holds important public health and policy implications; there are important implications because a majority of rape-related pregnancies occur among adolescents and result from assault by a related perpetrator and are closely linked with family and domestic violence. This concept is further supported by the fact that > 40% of rape-related pregnancies resulted from multiple assaults rather than from a single attack and thus occurred in a setting of ongoing violence or abuse [32].

In order to make a disclosure, a child victim must make public an event that probably involves some combination of personal shame, fear, or anticipation of negative consequences like blame, disbelief, and stigmatization [12]. In addition, in cases of intrafamilial abuse, victims often experience substantial emotional conflict about making disclosures that implicate parents or other loved ones, and may fear family disruption [33]. These factors can be difficult to overcome and, as a result, children may not make immediate disclosures following sexual victimizations. Intrafamilial CSA is less likely to be reported to police that extrafamilial CSA. Disclosures made in childhood, particularly disclosures of incest, are more negatively received by others than are disclosures made in adulthood [34, 35]. Most disclosures during childhood are made to parents, who are likely to be extremely upset by learning about incest [36] and that disclosure itself is not necessarily conducive to healing [32].

## Conclusions

Incest is both more common and more severe in stepparent families, but reported cases are only the very tip of the iceberg and may greatly under-represent actual population rates. Thus, more effort is required to hold the perpetrators accountable and restore the life of the victims.

## Abbreviations

CSA: child sexual abuse
UN: United Nations

## Authors’ information

YoMA is an assistant professor of public health at Wolaita Sodo University, College of Health Sciences and Medicine, School of Public Health, Sodo, Ethiopia.

BTM is senior lecturer of reproductive health at Jigjiga University, College of Medicine and Health Science, Jigjiga, Ethiopia.

YiMA is a medical doctor at Jimma University, College of Public Health and Medical Sciences, Department of Pediatrics, Jimma, Ethiopia.

## References

[1] Schneider EM. Domestic violence law reform in the twenty-first century: looking back and looking forward. Fam Law Q. 2008;42(3):353–63.

[2] Adinew YM, Hagos MA. Sexual violence against female university students in Ethiopia. BMC Int. Health Hum. Rights. 2017;17:19. 10.1186/s12914-017-0127-1. PMC5525286 28738807

[3] Koohi-Kamali, Feridoon, Intrahousehold Inequality and Child Gender Bias in Ethiopia (October 1, 2008). World Bank Policy Research Working Paper Series, Vol. , pp. -, 2008. Available at SSRN: https://ssrn.com/abstract=1293168

[4] Russell DEH. The incidence and prevalence of intrafamilial and extrafamilial sexual abuse of female children. Child Abuse Negl. 1983;7:133–46. 10.1016/0145-2134(83)90065-0 6605793

[5] Turner HA, Finkelhor D, Ormrod R. Family structure variations in patterns and predictors of child victimization. Am J Orthopsychiatry. 2007;77(2):282–95. 10.1037/0002-9432.77.2.282. 17535126

[6] Russell DEH. The prevalence and seriousness of incestuous abuse: stepfathers vs. biological fathers. Child Abuse Negl. 1984;8(1):15–22. 10.1016/0145-2134(84)90045-0. 6609753

[7] Saunders BE, Villoponteaux LA, Lipovsky JA, *et al.* Child sexual assault as a risk factor for mental disorders among women: a community survey. J. Interpers. Violence. 1992;7:189–204.

[8] World Health Organization (WHO). Guidelines for medico-legal care for victims of sexual violence. Geneva: World Health Organization; 2003.

[9] Bryer JB, Nelson BA, Miller JB, Krol PA. Childhood sexual and physical abuse as factors in adult psychiatric illness. Am J Psychiatr. 1987;144:1426–30. 10.1176/ajp.144.11.1426 3674223

[10] Russell DEH. The secret trauma: incest in the lives of girls and women. New York: Basic Books; 1986.

[11] Siibert MH, Pines AM. Sexual child abuse as an antecedent to prostitution. Child Abuse & Neglect. 1981;5:407–11.

[12] Browne A, Finkelhor D. Impact of child sexual abuse: a review of the research. Psychol Bull. 1986;99:66–77. 3704036

[13] Koss MP, Heslet L. Somatic consequences of violence against women. Arch Fam Med. 1992;1:53–9. 10.1001/archfami.1.1.53 1341588

[14] Finkelhor D. The sexual abuse of children: current research reviewed. Psychiatr Ann. 1987;17:233–41.

[15] Tsai M, Feldman-Summers S, Edgar M. Childhood molestation: variables related to differential impacts on psychosexual functioning in adult women. J Abnorm Psychol. 1979;88:401–17. 10.1037//0021-843x.88.4.407 479463

[16] Herman J, Russell D, Trocki K. Long-term effects of incestuous abuse in childhood. Am J Psychiatr. 1986;143:1293–6. 10.1176/ajp.143.10.1293 3766794

[17] Dinwiddie S, Heath AC, Dunne MP, Bucholz KK, Madden PA, Slutske WS, *et al.* Early sexual abuse and lifetime psychopathology: A co-twin-control study. Psychol Med. 2000;30(1):41–52. 10.1017/s0033291799001373 10722174

[18] Putnam FW. Ten-year research update review: child sexual abuse. J Am Acad Child Adolesc Psychiatry. 2003;42(3):269–78. 10.1097/00004583-200303000-00006 12595779

[19] Weiss EL, Longhurst JG, Mazure CM. Childhood sexual abuse as a risk factor for depression in women: psychosocial and neurobiological correlates. Am J Psychiatr. 1999;156(6):816–28. 10.1176/ajp.156.6.816 10360118

[20] Duncan RD, Saunders BE, Kilpatrick DG, Hanson RF, Resnick HS. Childhood physical assault as a risk factor for PTSD, depression, and substance abuse: findings from a national survey. Am J Orthopsychiatry. 1996;66(3):437–48. 10.1037/h0080194 8827267

[21] Miller MW, Resick PA. Internalizing and externalizing subtypes in female sexual assault survivors: implications for the understanding of complex PTSD. Behav Ther. 2007;38(1):58–71. 10.1016/j.beth.2006.04.003 PMC2977529 17292695

[22] Walsh K, Danielson CK, McCauley JL, Saunders BE, Kilpatrick DG, Resnick HS. National prevalence of posttraumatic stress disorder among sexually revictimized adolescent, college, and adult household-residing women. Arch Gen Psychiatry. 2012;69(9):935–42. 10.1001/archgenpsychiatry.2012.132 PMC3474859 22566561

[23] Daray FM, *et al.* The independent effects of child sexual abuse and impulsivity on lifetime suicide attempts among female patients. Child Abuse Negl. 2016;58:91–8. 10.1016/j.chiabu.2016.06.011 27352091

[24] Brodsky BS, Oquendo M, Ellis SP, Haas GL, Malone KM, Mann JJ. The relationship of childhood abuse to impulsivity and suicidal behavior in adults with major depression. Am J Psychiatr. 2001;158(11):1871–7. 10.1176/appi.ajp.158.11.1871 11691694

[25] Gladstone GL, Parker GB, Mitchell PB, Malhi GS, Wilhelm K, Austin MP. Implications of childhood trauma for depressed women: an analysis of pathways from childhood sexual abuse to deliberate self-harm and re-victimization. Am J Psychiatr. 2004;161(8):1417–25. 10.1176/appi.ajp.161.8.1417 15285968

[26] Ullman SE, Brecklin LR. Sexual assault history and suicidal behavior in a national sample of women. Suicide Life Threat Behav. 2002;32(2):117–30. 10.1521/suli.32.2.117.24398 12079028

[27] Bagley C, Ramsay R. Sexual abuse in childhood: psychosocial outcomes and implications for social work practice. Journal of Social Work and Human Sexuality. 1986;4:33–47.

[28] Stein JA, Golding JM, Siegel JM, Bumam MA, Sorenson SB. Long-term psychological sequelae of child sexual abuse: The Los Angeles Epidemiologic Catchment Area Study. In: Wyatt GE, Powell GJ, editors. Lasting effects of child sexual abuse. Newbury Park: Sage; 1988. p. 135–54.

[29] Brunngraber LS. Father-daughter incest: Immediate and long-term effects of sexual abuse. Advances in Nursing Science. 1986;8:15–35. 3089132

[30] Sedney MA, Brooks B. Factors associated with a history of childhood sexual experience in a nonclinical female population. J Am Acad Child Psychiatry. 1984;23:215–8. 10.1097/00004583-198403000-00015 6715743

[31] Peters SD. Child sexual abuse and later psychological problems. In: Wyatt GE, Powell GJ, editors. Lasting effects of child sexual abuse. Newbury Park: Sage; 1988. p. 101–17.

[32] Holmes MM, Resnick HS, Kilpatrick DG, Best CL. Rape-related pregnancy: Estimates and descriptive characteristics from a national sample of women. Am J Obstet Gynecol. 1996;175(2) 10.1016/s0002-9378(96)70141-2 8765248

[33] Lawson L, Chaffin M. False negatives in sexual abuse disclosure interviews: incidence and influence of caretaker’s belief in abuse in cases of accidental abuse discovery by diagnosis of STD. J. Interpers. Violence. 1992;7:532–42.

[34] Lamb S, Edgar-Smith S. Aspects of disclosure: mediators of outcome of childhood sexual abuse. J. Interpers. Violence. 1994;9:307–26.

[35] Roesler TA. Reactions to disclosure of childhood sexual abuse: the effect on adult symptoms. J Nerv Ment Dis. 1994;182:618–24. 10.1097/00005053-199411000-00004 7964669

[36] Roesler TA, Wind TW. Telling the secret: adult women describe their disclosures of incest. J. Interpers. Violence. 1994;9:327–38.

